# Pulsed electromagnetic fields potentiate the paracrine function of mesenchymal stem cells for cartilage regeneration

**DOI:** 10.1186/s13287-020-1566-5

**Published:** 2020-02-03

**Authors:** Dinesh Parate, Nurul Dinah Kadir, Cenk Celik, Eng Hin Lee, James H. P. Hui, Alfredo Franco-Obregón, Zheng Yang

**Affiliations:** 1 Department of Surgery, National University of Singapore, Singapore, 119228 Singapore; 2 Biolonic Currents Electromagnetic Pulsing Systems Laboratory, BICEPS, National University of Singapore, Singapore, Singapore; 3 Department of Orthopaedic Surgery, Yong Loo Lin School of Medicine, National University of Singapore, NUHS Tower Block, Level 11, 1E Kent Ridge Road, Singapore, 119288 Singapore; 4 Tissue Engineering Program, Life Sciences Institute, National University of Singapore, DSO (Kent Ridge) Building, #04-01, 27 Medical Drive, Singapore, 117510 Singapore; 5 Institute for Health Innovation & Technology, iHealthtech, National University of Singapore, Singapore, Singapore

**Keywords:** Pulse electromagnetic fields, Mesenchymal stem cells, Cartilage, Paracrine

## Abstract

**Background:**

The mesenchymal stem cell (MSC) secretome, via the combined actions of its plethora of biologically active factors, is capable of orchestrating the regenerative responses of numerous tissues by both eliciting and amplifying biological responses within recipient cells. MSCs are “environmentally responsive” to local micro-environmental cues and biophysical perturbations, influencing their differentiation as well as secretion of bioactive factors. We have previously shown that exposures of MSCs to pulsed electromagnetic fields (PEMFs) enhanced MSC chondrogenesis. Here, we investigate the influence of PEMF exposure over the paracrine activity of MSCs and its significance to cartilage regeneration.

**Methods:**

Conditioned medium (CM) was generated from MSCs subjected to either 3D or 2D culturing platforms, with or without PEMF exposure. The paracrine effects of CM over chondrocytes and MSC chondrogenesis, migration and proliferation, as well as the inflammatory status and induced apoptosis in chondrocytes and MSCs was assessed.

**Results:**

We show that benefits of magnetic field stimulation over MSC-derived chondrogenesis can be partly ascribed to its ability to modulate the MSC secretome. MSCs cultured on either 2D or 3D platforms displayed distinct magnetic sensitivities, whereby MSCs grown in 2D or 3D platforms responded most favorably to PEMF exposure at 2 mT and 3 mT amplitudes, respectively. Ten minutes of PEMF exposure was sufficient to substantially augment the chondrogenic potential of MSC-derived CM generated from either platform. Furthermore, PEMF-induced CM was capable of enhancing the migration of chondrocytes and MSCs as well as mitigating cellular inflammation and apoptosis.

**Conclusions:**

The findings reported here demonstrate that PEMF stimulation is capable of modulating the paracrine function of MSCs for the enhancement and re-establishment of cartilage regeneration in states of cellular stress. The PEMF-induced modulation of the MSC-derived paracrine function for directed biological responses in recipient cells or tissues has broad clinical and practical ramifications with high translational value across numerous clinical applications.

**Electronic supplementary material:**

The online version of this article (10.1186/s13287-020-1566-5) contains supplementary material, which is available to authorized users.

## Background

Articular cartilage is an avascular tissue with limited intrinsic capacity for regeneration. In combination with the limitations of existing treatment modalities, joint injuries often deteriorate with time into articular joint disease [1, 2]. Mesenchymal stem cells (MSCs), with their capacity for expansion and demonstrated multipotency for a variety of tissue lineages, have been championed as a promising cell source for the repair and regeneration of many degenerative, inflammatory, or autoimmune diseases [3]. Nevertheless, despite their anticipated, but at times not substantiated, potential, MSC-based strategies have often fallen short of initial expectations [4]. Although the potential applicability of MSCs for cartilage repair was initially postulated based on their ability to differentiate into chondrocytes and to participate in the formation of tissue, it has become increasingly evident that part of the reparative value of MSCs is attributed to their paracrine manner of developmental entrainment [5]. In non-contact co-culture experiments between MSCs and primary chondrocytes, improvements in chondrocyte proliferation, phenotype maintenance, and enhanced matrix synthesis were shown to be credited to MSC-derived trophic factors [6, 7]. A paracrine role of MSCs was further supported by preclinical studies showing that intra-articular injection of MSCs prevented the development of post-traumatic arthritis and promoted cartilage regeneration in damaged joints [8], as well as improved clinical outcomes and indices of cartilage repair in second-look arthroscopy [9].

The spectrum of trophic factors released by MSCs is collectively referred to as the secretome. Via the combined actions of its plethora of biologically active factors, the MSC secretome is capable of establishing a regenerative microenvironment for the repair of injured tissues by both eliciting and amplifying biological responses within recipient cells. Compositional analyses of the secretome using mass spectrometry, next-generation sequencing, or lipid profiling have identified cytokines and growth factors with diverse biological properties that have been implicated in a vast array of cellular processes ranging from cell activation and proliferation to differentiation. Components of the secretome have also been ascribed key roles in critical aspects of tissue regeneration such as promoting angiogenesis, inhibiting apoptosis, immunomodulation, anti-inflammation, and stem cell homing to sites of injury [10–12]. In particular, MSC exosomes were demonstrated to possess therapeutic potential for cartilage repair in osteochondral defects [13, 14] as well as protection against cartilage and bone degradation in in vivo models of osteoarthritis [15, 16].

The function of the MSC secretome is activated by local micro-environmental cues that modulate MSC differentiation as well as that of subsidiary tissues [17, 18]. Changes in growth factors/cytokines [19, 20], oxygen tension [21, 22], or environmental mechanical cues arising from the extracellular matrix [23, 24] or substrate stiffness [25, 26] have been shown to directly influence MSC paracrine activity. Scaffolds of distinct composition and cytoarchitecture influence how the cellular mechanotransduction machinery translates environmental mechanical and chemical cues into transcriptional and paracrine responses. Moreover, growing MSCs as 3D spheroids encourages cell-cell interactions that upregulate paracrine activities with anti-inflammatory and angiogenic properties [27–29]. Furthermore, diverse forms of mechanical stimulation such as shear stress, tensile stress, and compression have been shown to promote the production of reactive oxygen species (ROS) [30], as well as alter the secretome profile of MSCs [31, 32]. Accordingly, activation of mitochondrial respiration is known to activate the muscular secretome [33].

**Fig. 1:**
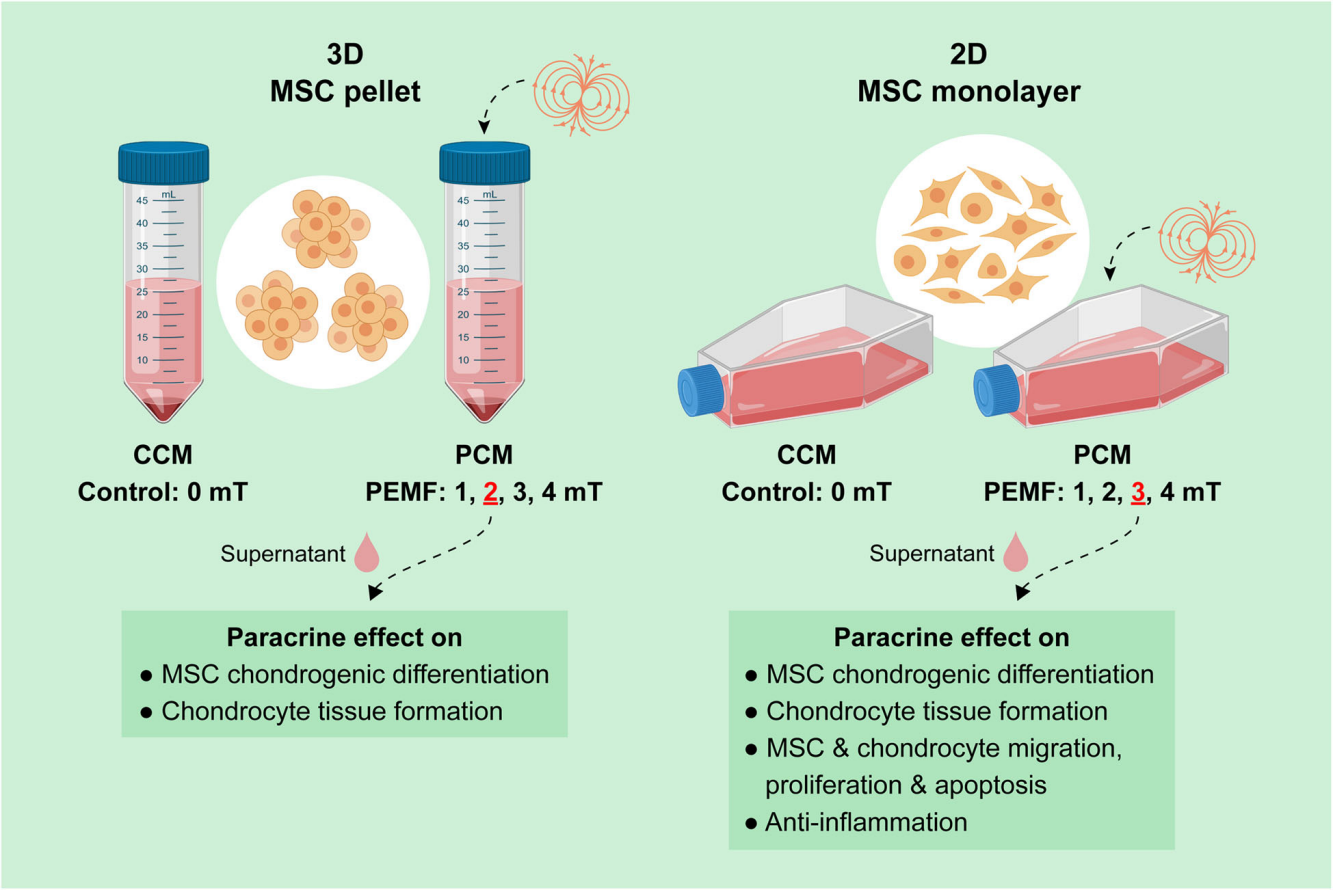
Schematic illustration of the generation and functional analysis of the CM from MSCs subjected to either 3D or 2D culturing platforms, with (PCM) or without (CCM) PEMF exposure

We have previously shown that pulsed electromagnetic fields (PEMFs) activate calcium-permeable transient receptor potential (TRP) channels, promoting both in vitro chondrogenesis [34] and myogenesis [35] by activating a calcium-mitochondrial transcriptional and epigenetic axes governing survival and development [35]. PEMF-induced MSC differentiation has been correlated with increased expression of TGFβ and BMP2 [36–38]. Moreover, the expression and paracrine action of these growth factors was also demonstrated in electrically driven MSC chondrogenesis [39]. PEMF exposure has also been shown to exert anti-inflammatory effects by upregulating A2A and A3ARs, thereby mitigating the expression of pro-inflammatory cytokines [40, 41]. This is supported by studies showing PEMF inhibition of the PGE2 and cycloxigenase-2 (COX-2) pathways, reducing the expression of pro-inflammatory cytokines (IL-6, IL-8) while augmenting anti-inflammatory factors (cAMP, IL-10) in synovial fibroblasts from bovine and osteoarthritic patients [42–44]. Here, we provide evidence that the previously described pro-chondrogenic of PEMF stimulation [34] can be largely attributed to its modulation of the MSC secretome (Fig. 1). PEMF stimulation was shown to modulate the chondrogenic, chemotactic, anti-inflammatory, and antiapoptotic activities of the MSC secretome in the form of conditioned medium (CM) harvested from PEMF-exposed MSCs. PEMF stimulation, via its effects over MSC paracrine signaling, might hence represent a manner of promoting regeneration in an inflammatory joint environment.

## Methods

### PEMF exposure system

The PEMF device used in this study has been previously described [34, 35]. Briefly, the device produces spatially homogeneous, time-varying magnetic fields, consisting of barrages of 20 × 150 μs on and off pulses for 6 ms repeated at a frequency of 15 Hz. The magnetic flux density rose to predetermined maximal level within ~ 50 μs (~ 17 T/s) when driving field amplitudes between 0.5 and 4 mT. Unless explicitly noted, all samples were exposed once for 10 min. All PEMF-treated samples were compared to time-matched control samples (0 mT) that were manipulated in exactly the same manner as experimental samples, including placement into the PEMF-generating apparatus for the designated time, except that the apparatus was not set to generate a magnetic field.

### Human bone marrow MSC culture

Primary human mesenchymal stem cells (MSCs) were purchased from RoosterBio Inc. (Frederick, MD), supplied at passage 3. MSCs were further expanded in MSC High Performance Media (RoosterBio Inc.) at 37 °C in 5% CO_2_ atmosphere. The expanded MSCs were used between passages 5 and 6.

### Conditioned media (CM) from MSC in 3D and 2D culture platforms

Conditioned media (CM) was generated from 3D culture pellets as previously described [34]. Briefly, MSCs were expanded in expansion media until 70–80% confluency before being subjected to pellet formation (2.5 × 10^5^ cells per pellet) and left in expansion media overnight. The following day the expansion media was replaced with 0.5 ml of low-glucose DMEM (Life Technologies) media without FBS. PEMF treatment was applied for a duration of 10 min at 1, 2, 3, and 4 mT, or 2 mT for 30 min. CMs from pellet culture were collected 24 h post-PEMF exposure and pooled for later use.

To generate CM from 2D culture platforms, MSCs were cultured in T75 flasks at a seeding density of 1.5–2 × 10^5^ in expansion media. At 50–60% confluency, the expansion media was replaced with 10 ml of low-glucose DMEM media without FBS. PEMF exposure was applied at 1, 2, 3, and 4 mT for 10 min, or 2 mT for 30 min, and the CM generated (PCM) was collected at 24 h post-PEMF exposure, pooled, and used for further application and analysis. Control CM (CCM) from 2D and 3D culture platform was generated from similarly cultured MSC without PEMF treatment (0 mT).

In either culturing platform, CM was collected and subsequently concentrated 10× by high centrifugation force using a protein concentrator with a molecular weight cut-off of 3 kDa (Thermo Fisher Scientific, USA). In subsequent experiments, the concentrated CM was diluted 1:10 with low-glucose DMEM media without FBS prior to application to the experimental culture to achieve a final working strength of 1× CM.

### MSC chondrogenesis

Chondrogenic differentiation of MSCs was induced in 3D pellet cultures as previously described [45, 46]. Briefly, 2.5 × 10^5^ cells were centrifuged to form pellets and cultured in a chondrogenic differentiation medium containing high-glucose DMEM supplemented with 4 mM proline, 50 μg/mL ascorbic acid, 1% ITS-Premix (Becton-Dickinson, San Jose, CA), 1 mM sodium pyruvate, and 10^− 7^ M dexamethasone (Sigma Aldrich, St Louis, MO), for up to 7 or 21 days in the presence of 10 ng/mL of transforming growth factor-β3 (TGFβ3; R&D Systems, Minneapolis, MN). Streptomycin and penicillin were excluded from the chondrogenic differentiation media to avoid interference with TRPC channel gating [34, 35]. To study the chondrogenic potential of the MSC secretome, the chondrogenic media, in the absence or presence of TGFβ3, was supplemented with CM.

### Chondrocyte redifferentiation

Chondrocytes were isolated from pig (animal not directly involved in this study) articular cartilage following enzymatic digestion as previously described [47]. Isolated chondrocytes were expanded in low-glucose DMEM with 10% FBS without antibiotics and used at passage 1 for all the experiments. Chondrogenic redifferentiation was induced in 3D pellet cultures in the presence of 10 ng/mL of TGFβ3. Briefly, 2 × 10^5^ chondrocyte cells were centrifuged to form pellets and kept overnight in expansion media at 37 °C in 5% CO_2_ atmosphere. To study the chondrogenic potential of the MSC secretome for chondrocyte redifferentiation, the expansion media was replaced with chondrogenic media supplemented with the CM and cultured up to 7 or 21 days with medium change every 2–3 days.

### Cell migration

The migration of MSCs and chondrocytes in response to CM was assessed using a 24-well Transwell culture (8 μm pore size, Millipore, Germany). Briefly, MSCs (3 × 10^4^) or chondrocytes (5 × 10^4^) were suspended in 300 μl of low-serum culture medium (DMEM supplemented with 0.5% FBS (Life Technologies)) and placed into the upper chamber, and CM was added to the lower chambers of the Transwell culture, containing DMEM with 0.5% FBS. After 16 h, the upper surface of the Transwell filters was swabbed to remove cells. Cells on the underside of the filter, representing the migrated cells, were then fixed in 4% (v/v) paraformaldehyde and stained with hematoxylin and eosin (Sigma Aldrich). The cells in five randomly selected fields at 40× magnification were counted to indicate migrated cells.

### Inflammatory induction of MSCs and chondrocytes

MSCs or chondrocytes were plated at 1.5 × 10^4^ or 3 × 10^4^ cells/well, respectively, in a 24-well plate in DMEM containing 10% FBS. IL-1β (5 ng/ml; RnD systems) was added to the expansion media at 24 h after cell seeding to simulate inflammatory conditions. To investigate the inflammation modulatory effect of MSC secretome, CM was added to the culture 24 h after the induction of inflammation. MSCs or chondrocytes, induced with IL-1β, without subsequent CM treatment served as inflammation (positive) controls, whereas non-inflammation (negative) controls consisted of MSCs or chondrocytes without the addition of IL-1β and CM treatments. Cells and media were harvested 24 and 48 h post-supplementation with CM for RNA and *nitric oxide synthase* (NOS) analysis. NOS activity in the media was analyzed with a NOS assay kit (Abcam, USA). Real-time PCR analysis was performed on the harvested cells to assess the inflammation modulation as a result of the CM.

For post-chondrogenic inflammation induction, MSC pellets were administered IL-1β (5 ng/ml) for 24 h before being supplemented with CM. The inflammation modulatory effect of the CM with reference to MSC-derived chondrogenesis was investigated by real-time PCR analysis at day 7 of differentiation.

### Cell proliferation and apoptosis

To assess cell proliferation DNA was analyzed using Quant-iT™ PicoGreen™ dsDNA Assay Kit (Life Technologies) over a period of 3 days. For determination of antiapoptotic capacity of CM, MSCs or chondrocytes were seeded at 1.5 × 10^4^ or 3 × 10^4^ cells/well in a 24-well plate and treated with Staurosporin (200 nM, Sigma Aldrich) for 2 h in the presence of CM. The extent of apoptosis was indicated by Caspase 3/7 activity using a Caspase 3/7 assay kit (Promega, Singapore).

### Real-time PCR analysis

Total RNA was extracted using the RNeasy® Mini Kit (Qiagen, Germany). Reverse transcription was performed with 100 ng total RNA using iScript™ cDNA synthesis kit (Bio-Rad, USA). Real-time PCR was conducted using the SYBR® green assay on ABI Step One Plus Real-Time PCR System (Applied Biosystems, Life Technologies, USA). Real-time PCR program was set at 95 °C for 10 min, followed by 40 cycles of amplifications, consisting of a 15 s denaturation at 95 °C and a 1 min extension step at 60 °C. The human and porcine primer sequences used in this study are listed in Additional file 1: Table S1. The level of expression of the target gene, normalized to GAPDH, was then calculated using the 2^−ΔΔCt^ formula with reference to the undifferentiated MSC. Results were averaged from triplicate samples of two independent experiments.

### ECM and DNA quantification

Samples harvested were digested with 10 mg/mL of pepsin in 0.05 M acetic acid at 4 °C, followed by digestion with elastase (1 mg/mL). A Blyscan sulfated glycosaminoglycan (sGAG) assay kit (Biocolor Ltd., Newtownabbey, Ireland) was used to quantify sGAG deposition according to manufacturer’s protocol. Absorbance was measured at 656 nm, and sGAG concentration was extrapolated from a standard curve generated using a sGAG standard. Type II Collagen (Col 2) content was measured using a captured enzyme-linked immunosorbent assay (Chondrex, Redmond, WA). Absorbance at 490 nm was measured and the concentration of Col 2 was extrapolated from a standard curve generated using a Col 2 standard. Values for sGAG and Col 2 content obtained were normalized to the total DNA content of respective samples, measured using Picogreen dsDNA assay (Molecular Probes, OR, USA). Quadruplicates of each group were analyzed from two independent experiments.

### Secretome analysis

A RayBio fluorescent antibody array (Genomax Technologies, SG) was customized for analyzing the secretome of MSC. The CM of 2D cultured MSCs without (0 mT) and with PEMF exposure at 3 mT for 10 min were concentrated 10× using a protein concentrator with a molecular weight cut-off of 3 kDa (Thermo Fisher Scientific, USA). The staining of the arrays was performed according to the manufacturer’s protocol. Images were acquired using a GenePix 4000B microarray scanner and analyzed with GenePix Pro software (Molecular Devices, USA) for the relative fluorescent intensities of the customized protein targets.

### Statistical analysis

All experiments were performed in biological replicates (*n* = 3) and results reported as mean ± standard deviation (SD). Statistical analysis was carried out by Student’s *t* test for comparison between two groups using the Microsoft Excel software. The level of significance was set at *p* < 0.05. All quantitative data reported were averaged from at least two independent experiments.

## Results

### Chondrogenic potential of PEMF-conditioned media generated from MSCs in 3D culture

**Fig. 2:**
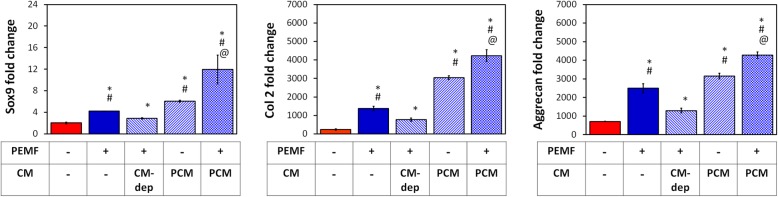
Brief PEMF exposure stimulates MSC-derived paracrine activity to promote chondrogenesis. MSCs in 3D pellet cultures undergoing chondrogenesis were subjected to PEMFs at either 0 or 2 mT for 10 min. The conditioned media generated after 24 h of PEMF exposure (PCM) was either replaced with age-matched media from unexposed sister cultures (CM-dep; CM-deprived), or transferred to age-matched exposed or unexposed sister cultures. Real-time PCR analysis of cartilaginous markers were performed after 7 days of differentiation and normalized to GAPDH. Results were presented as fold-changes relative to the level in undifferentiated MSCs. Data shown are means ± SD, *n* = 6 from 2 independent experiments. * denotes significance differences compared to non-PEMFed controls (red bars); # denotes significance differences compared to CM-deprived (CM-dep) condition; @ denotes significance differences compared to treatment with PCM alone

We have previously shown that MSCs in pellet culture (3D) exhibited an enhancement in chondrogenic induction when exposed to PEMFs at a discrete efficacy window of 2 mT applied once for 10 min [34]. Here we show that the MSC secretome contributes to the chondrogenic potential of PEMF exposure. The characteristic PEMF-induced upregulations of Col 2, Aggrecan, and Sox9 were attenuated by the removal of PCM and its replacement with age-matched media harvested from naïve (CM-deprived; CM-dep) unexposed sister cultures 24 h following PEMF exposure (Fig. 2), serving as a manner to selectively deprive cells of PEMF-mediated paracrine stimulation, while leaving other collateral PEMF-dependent responses intact. On the other hand, PEMF-induced chondrogenic induction could be transferred to naïve MSCs with the transfer of CM harvested from PEMF-treated MSCs (PCM) 24 h after exposure. Moreover, PCM acted synergistically with the effects of direct PEMF exposure. The results indicate that the chondrogenic attributes of PEMF exposure is partially mediated through the paracrine activity of the MSC secretome.

**Fig. 3:**
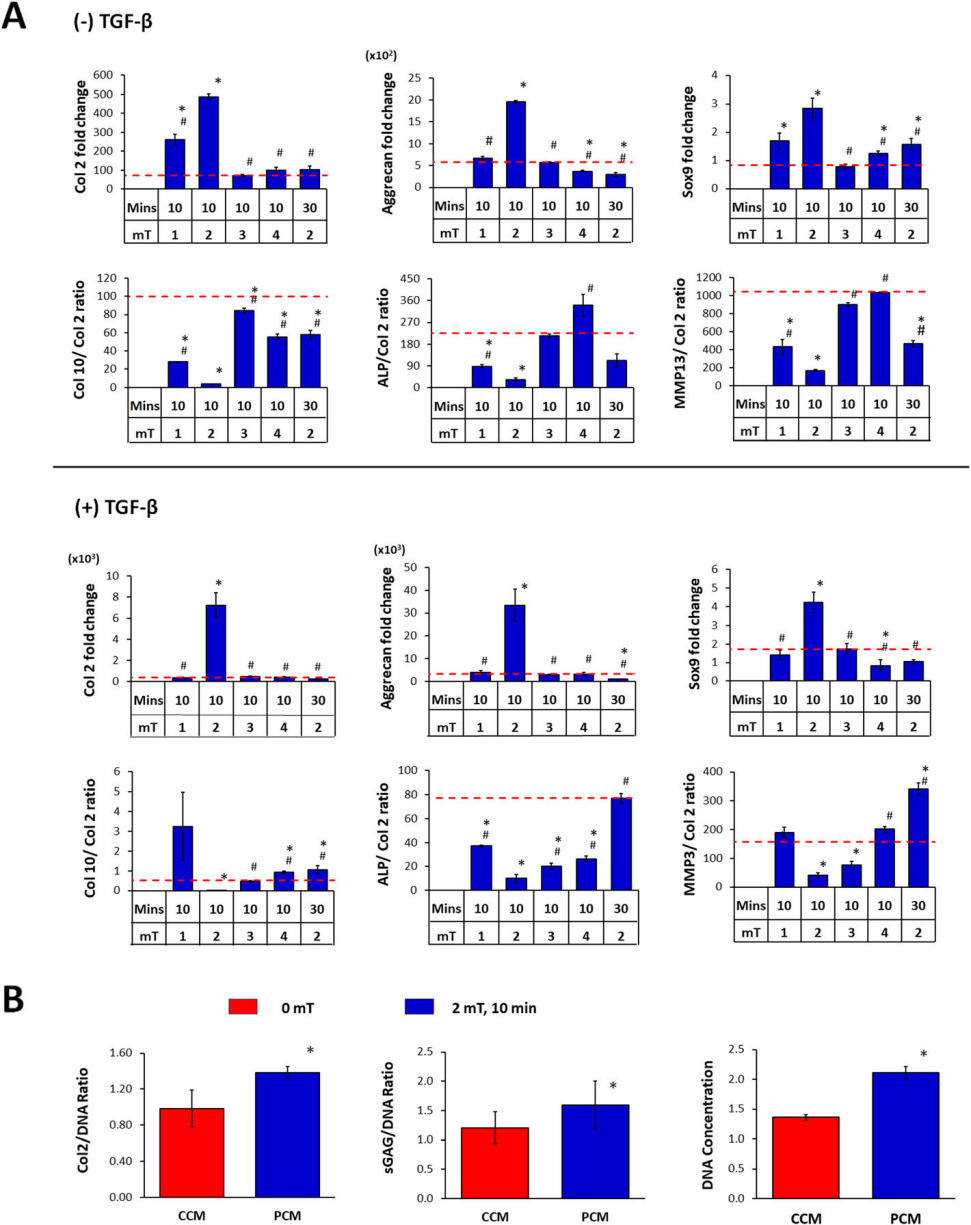
Effect of PEMF-induced conditioned media (CM) from 3D cultured MSCs on chondrogenic differentiation of MSCs. MSCs in 3D culture were subjected to PEMF exposure at different intensities and durations. The generated CM was collected 24 h post-PEMF exposure and tested for chondrogenic effect over naive (unexposed) MSC pellet cultures undergoing chondrogenic differentiation in the absence or presence of TGFβ. **a** Real-time PCR analysis of cartilaginous and hypertrophic marker expression after 7 days of differentiation normalized to GAPDH and presented as fold-changes relative to levels in undifferentiated MSCs. The expression of hypertrophic markers was presented as the ratio to Col 2 expression. **b** Quantification of cartilaginous extracellular matrix macromolecules generated by the differentiated MSCs (+TGFβ) after 21 days of differentiation. Data shown represent means ± SD, *n* = 6 from 2 independent experiments. * denotes significance differences compared to non-PEMFed (CCM, 0 mT) controls (red dash lines or red bars). # denotes significance differences compared to PEMFed CM (PCM) generated at 2 mT, 10 min

MSC pellet cultures were exposed to PEMFs of varying amplitudes and durations and the generated PCMs were transferred to naïve (unexposed) MSC pellet cultures undergoing chondrogenic induction. PCM generated from MSC pellets exposed at 2 mT for 10 min produced the greatest induction of chondrogenic markers (Col 2, aggrecan, Sox9) in naïve MSC pellets (Fig. 3a), matching our previously described EMF efficacy window [34]. Conversely, the expression ratios of type X collagen (Col 10), alkaline phosphatase (ALP) and matrix metallopeptidase 13 (MMP13) to Col 2, indices of chondrogenic hypertrophy, were most strongly suppressed by PCM generated at the peak of the efficacy window (2 mT for 10 min).

TGFβ3 is commonly used to facilitate chondrogenic induction [45, 46]. Notably, PCM generated at 2 mT for 10 min was capable of promoting chondrogenesis in the absence of TGFβ3 by four to fivefold. In the presence of 10 ng/ml TGFβ3, chondrogenic induction was further enhanced by PCM exhibiting increases of > 10-fold for both Col 2 and aggrecan. Sox9 expression levels were more modestly enhanced with PCM generated at 2 mT for 10 min in the absence (~ 3-fold) or presence (~ 4-fold) of TGFβ. By contrast, PCM harvested from 3D MSC cultures that were exposed to PEMFs of greater amplitudes or longer durations did not exhibit any significant effects relative to pellets administered control CM (CCM, 0 mT). The inhibition of chondrogenic hypertrophy by PCM harvested at peak PEMF amplitude (2 mT) was also preserved in the presence of TGFβ3 (Fig. 3a).

The chondrogenic enhancement afforded by PCM administration at the transcriptional level was further corroborated at the protein level, whereby the PCM obtained from MSC 3D pellet cultures following exposure to 2 mT PEMFs produced significant increases in both Col 2 and sGAG proteins with an associated increase in cellular DNA content relative to CCM (Fig. 3b).

### Chondrogenic potential of PCM generated from MSCs in 2D culture

**Fig. 4:**
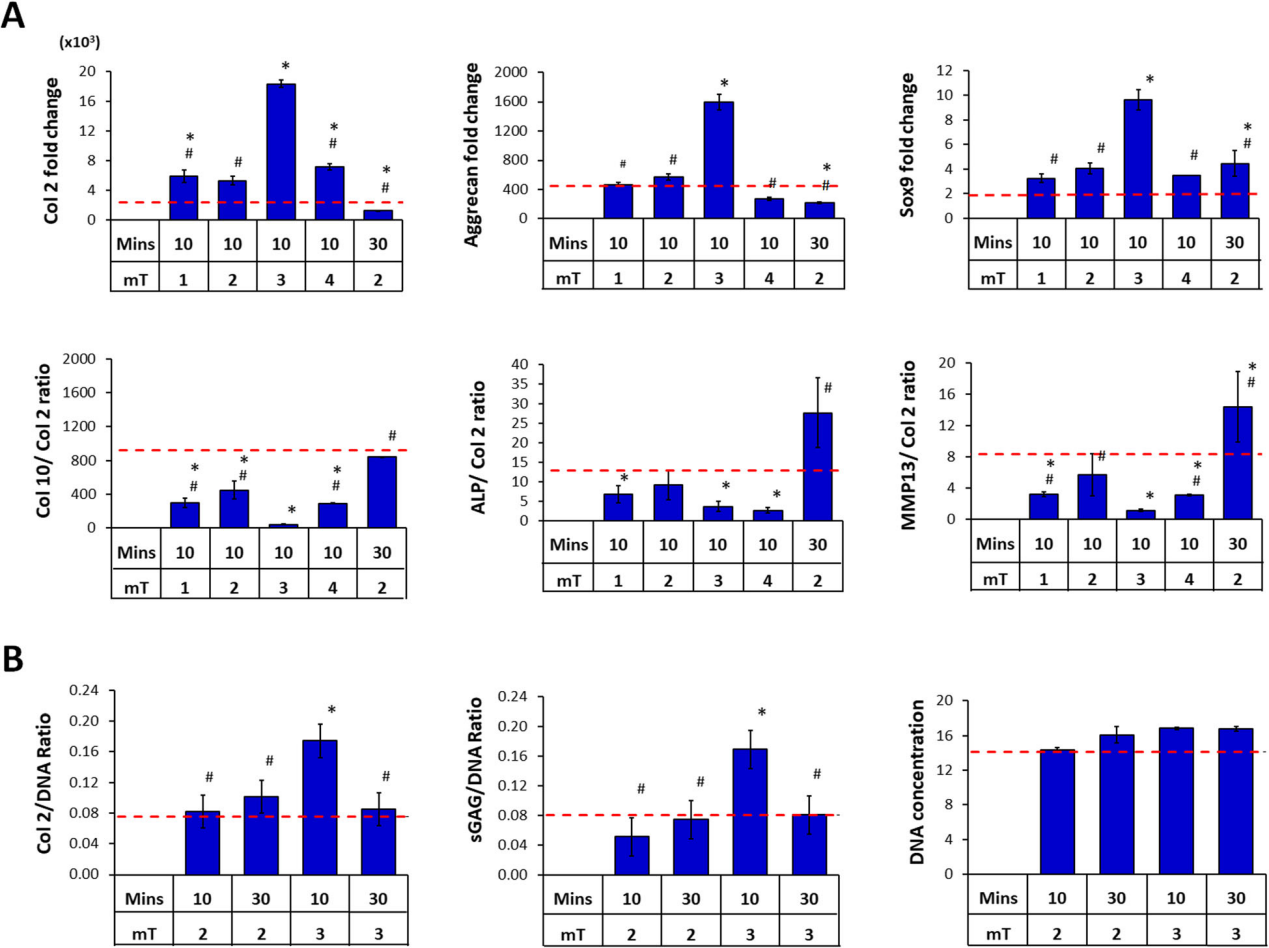
Effects of PEMF-induced conditioned media (CM) harvested from 2D cultures of MSCs over chondrogenic differentiation of MSCs. CM was collected 24 h post-PEMF exposure and tested for chondrogenic effect over naive (unexposed) MSC pellet cultures undergoing chondrogenic differentiation in the presence of TGFβ. **a** Real-time PCR analysis of cartilaginous and hypertrophic marker expression after 7 days of differentiation normalized to GAPDH and presented as fold-change relative to levels in undifferentiated MSCs. Expression of hypertrophic markers was presented as ratio to Col 2 expression. **b** Quantification of cartilaginous extracellular matrix macromolecules generated by differentiated MSCs after 21 days of differentiation. Data shown are means ± SD, *n* = 6 from 2 independent experiments. * denotes significance differences compare to non-PEMFed CM (0 mT) controls (red dash lines). # denotes significance differences compared to PEMFed CM (PCM) generated at 3 mT, 10 min exposure

It is well established that the availability and nature of cellular-substrate interactions influence the paracrine activity of MSCs [18, 23, 48]. We next assessed the chondrogenic potential of PCM harvested from MSCs grown in 2D cultures. MSCs cultured on the surface of tissue culture dishes were exposed to PEMF amplitudes ranging from 0 to 4 mT for single 10 min exposures and the generated CMs were then transferred to naïve (unexposed) MSC pellet cultures undergoing chondrogenic induction in the presence of TGFβ. PCM harvested from MSCs exposed to 3 mT for 10 min produced the greatest upregulations of Col 2 (~ 17-fold) and aggrecan (~ 4-fold) expression in naïve MSC pellets, whereas PCMs generated from MSCs exposed at PEMF amplitudes other than 3 mT or for longer exposures (30 min) produced no additional chondrogenic enhancement relative to MSC pellets treated with control CM (CCM, 0 mT) (Fig. 4a). As with 3D MSC-generated PCM, downregulations in the expression of the hypertrophic markers Col 10, ALP, and MMP13 were most apparent with peak PCM (3 mT) compared to CCM (Fig. 4a). The chondrogenic enhancement apparent at the transcriptional level with PCM generated from 2D MSCs exposed to 3 mT for 10 min was also corroborated at the protein level, with a twofold increase in Col 2 and sGAG deposition compared to non-PEMFed CCM (Fig. 4b). Accordingly, no increase in ECM deposition was detected with PCM generated at off peak conditions.

### Effects of PCM on chondrocytes

**Fig. 5:**
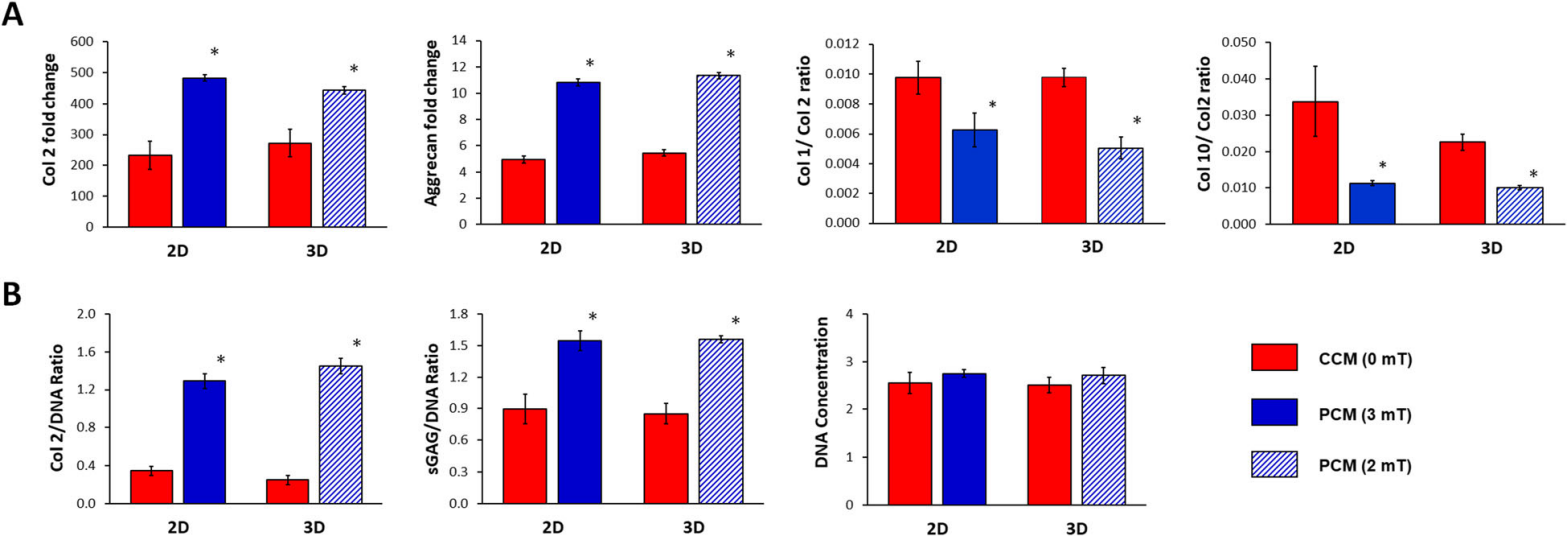
Effects of PEMF-induced conditioned medium (CM) on chondrocyte redifferentiation. CM was generated from MSCs cultured on 2D and 3D platforms with and without PEMF exposure at 3 mT and 2 mT, respectively, or unexposed. CM collected 24 h post-PEMF exposure was administered to chondrocyte pellet cultures. **a** Real-time PCR analysis of cartilaginous marker expression after 7 days of redifferentiation was normalized to GAPDH and presented as a fold-change relative to the level expressed in day 0 chondrocytes. Col 1 and Col 10 expressions are shown as ratios relative to Col 2 expression. **b** Quantification of cartilaginous extracellular matrix macromolecules generated by chondrocytes after 21 days of redifferentiation. Data shown represent means ± SD, *n* = 6 from 2 independent experiments. * denotes significant increases compare to respective non-PEMFed CM (CCM, 0 mT) (red solid bars)

We next investigated the chondrogenic effects of the MSC-PCM on chondrocyte redifferentiation. Chondrocytes as pellet cultures in the presence of TGFβ were treated with PCM generated from MSCs cultured in either 2D or 3D platforms exposed at their respective peak amplitudes of 3 mT and 2 mT. Both PCMs better sustained the chondrogenic phenotype in redifferentiating chondrocytes, relative to their respective CCM. Increased expression of Col 2 and aggrecan (1.5 to 2-fold) was detected, whereas decreases in the expression of Col 1 and Col 10 were observed, relative to the respective CCM (Fig. 5a). The relative efficacy of either PCM at its associated peak amplitude and platform on primary chondrocytes was further corroborated at the protein level for both Col 2 (> 3-fold increase) and sGAG (~ 2-fold increase) production (Fig. 5b). The results suggest that PCMs harvested from either 2D or 3D MSC cultures at their respective peak amplitudes and exposure durations were capable of offsetting chondrocyte de-differentiation and promote the expression of hyaline cartilage markers.

### Effect of PCM on chondrocyte and MSC migration and proliferation

**Fig. 6:**
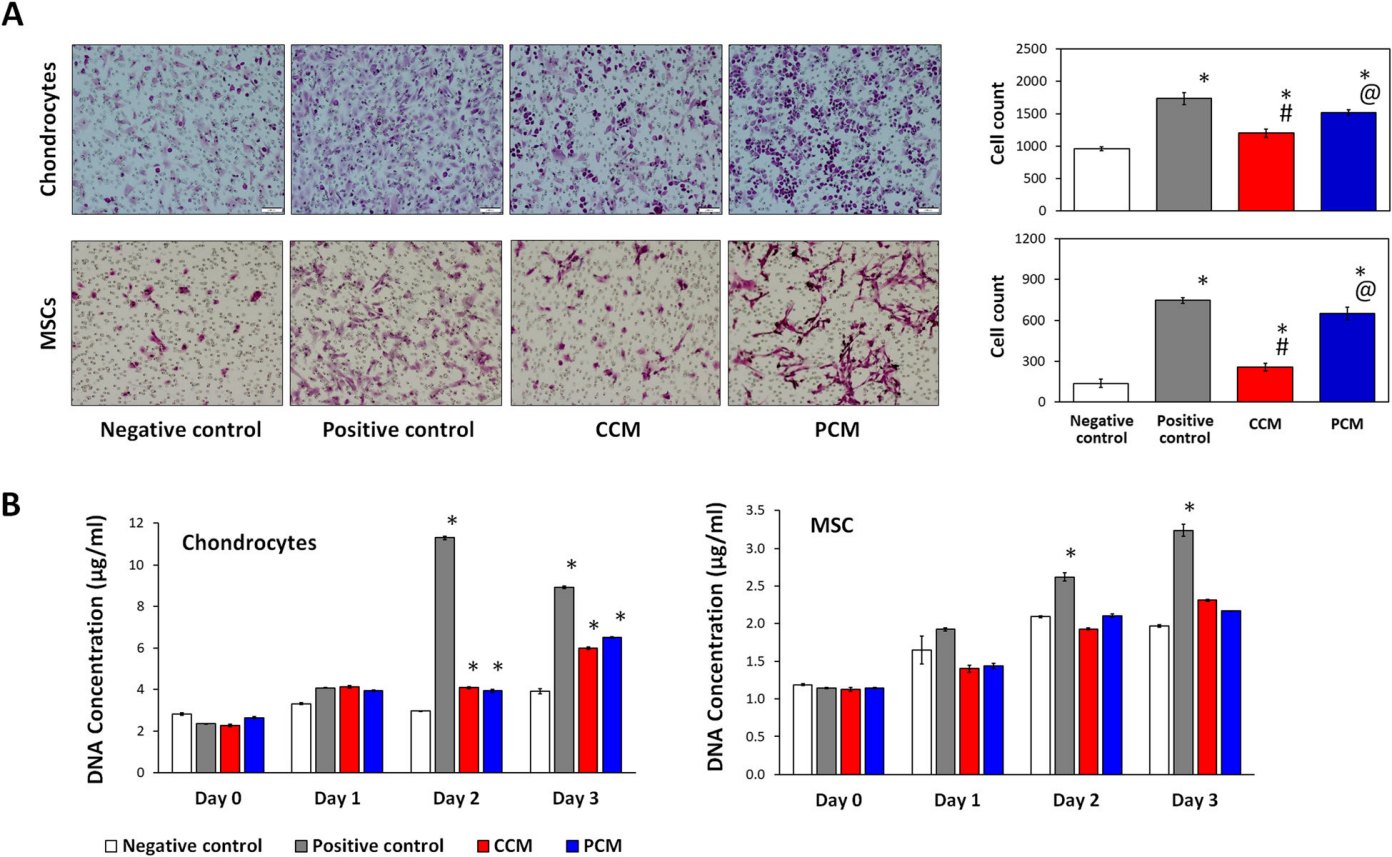
Effect of PEMF-induced conditioned medium (CM) on chondrocyte and MSC migration and proliferation. CM was derived from 2D cultured MSCs in response to 0 mT (CCM) or 3 mT (PCM) PEMF exposure. **a** Migration of chondrocytes or MSCs was analyzed using a transwell culture. Migrated cells were assessed by measuring the number of cells on the underside of the transwell filter after H&E staining. **b** Cell proliferation was determined by Picogreen DNA Assay. Data shown represent means ± SD, *n* = 6 from 2 independent experiments. * denotes significant difference compared to the negative control (Expansion media + 0.5% FBS); # denotes significant differences compared to positive controls (Expansion media + 10% FBS); @ denotes significant differences compared to the non-PEMFed CCM

We assessed if 2D MSC-generated PCM could affect the migration and proliferation of chondrocytes and MSCs, the two cell types localized in the articular joint environment that participate in cartilage regeneration [8, 49]. CCM and PCM enhanced cell migration of both chondrocytes and MSCs relative to respective negative controls (Fig. 6a). PCM, however, produced a further twofold and fourfold increase in migratory capacity for chondrocytes and MSCs, respectively, relative to the CCM, approaching the migration level observed for the positive controls (10% FBS). Chondrocyte proliferation was enhanced to similar magnitudes with either CCM or PCM, whereas MSC proliferation was largely unaffected by either CM under the presented culturing conditions (Fig. 6b). PCM thus appears to hold potential for enhancing MSC-derived paracrine-dependent chemotaxis of MSCs and chondrocytes.

### Anti-inflammatory effects of PCM on chondrocytes and MSCs

**Fig. 7:**
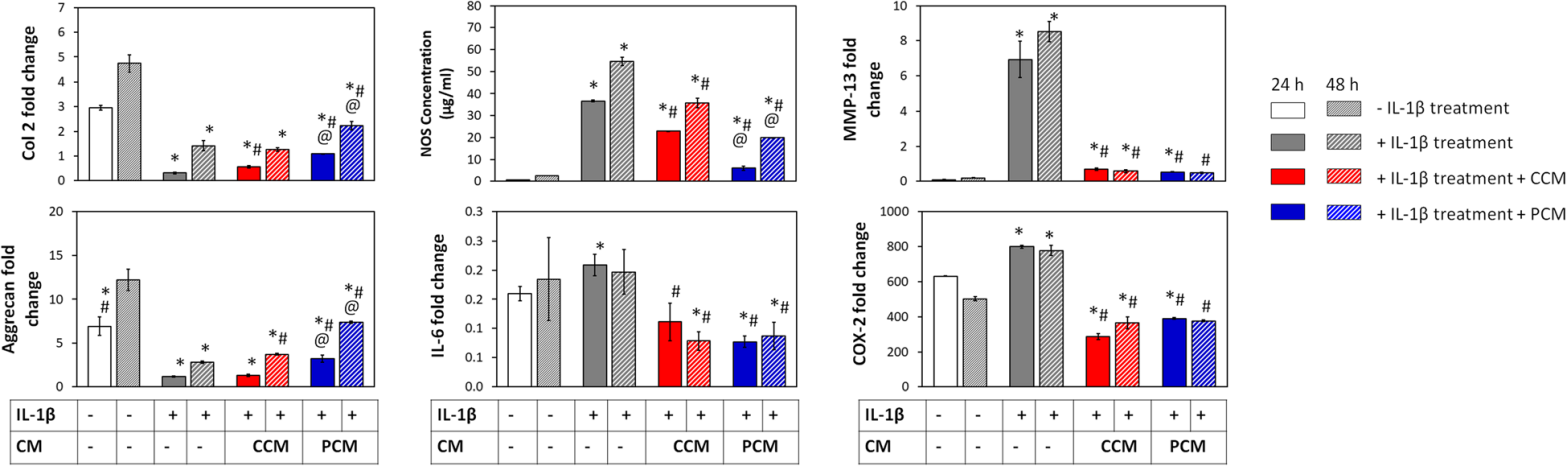
Effect of PEMF-induced conditioned medium (CM) on inflamed chondrocytes. Inflammation was induced in chondrocytes with 5 ng/ml IL-1β for 24 h. CCM or PCM was administered to chondrocytes 24 h post inflammation induction. Real-time PCR analysis of cartilaginous and inflammatory markers and NOS activity were performed at 24 h (plain bars) and 48 h (hatched bars) post-supplementation with CM and normalized to GAPDH, presented as a fold-change relative to the level in non-treated (day 0) chondrocytes. Data shown represent means ± SD, *n* = 6 from 2 independent experiments. * denotes significant differences compared to the non-inflamed controls (no IL-1β treatment); # denotes significant differences compared to the inflammation controls (IL-1β alone treatment) and @ denotes significant differences compared to respective CCM. Col 2 = type II collagen; COX-2 = cycloxigenase-2; IL-1β = interleukin-1β; IL-6 = interleukin-6; MMP-13 = metalloproteinase 13; NOS = *nitric oxide synthase*

The MSC secretome has been ascribed anti-inflammatory properties [14, 29]. We investigated the potential immunomodulatory attributes of 2D MSC-generated PCM over inflammation-induced chondrocytes. Primary chondrocytes treated with IL-1β exhibited attenuated expression of the ECM markers, Col 2 and aggrecan, coincident with an increased expression of the pro-inflammatory markers, IL-6, MMP13, and COX-2, as well as enhanced NOS activity (Fig. 7). Although NOS activity was suppressed by either PCM or CCM relative to the inflammatory control (IL-1β treatment), PCM exerted an additional ~ 2–3-fold greater suppression of NOS compared to CCM that was sustained throughout the 48-h examination period. Suppressions in the levels of IL-6 (> 1.5- and 2.5-fold), MMP-13 (10- and 13-fold), and COX-2 (> 2.5- and 2-fold) were also observed commencing at 24 h after administration of either CCM or PCM, respectively. Notably, chondrocytes exhibited elevated basal levels of IL-6 and COX-2 in the non-inflammation controls (no IL-1β treatment), indicating significant levels of resting inflammation in chondrocytes. Reinstated expression of chondrogenic markers (Col 2 and aggrecan) was observed following administration of the PCM at 24 and 48 h relative to the inflammatory control and CCM (Fig. 7) that, although were mitigated relative to the non-inflammatory controls, were showing a trend towards recovery at 48 h.

**Fig. 8:**
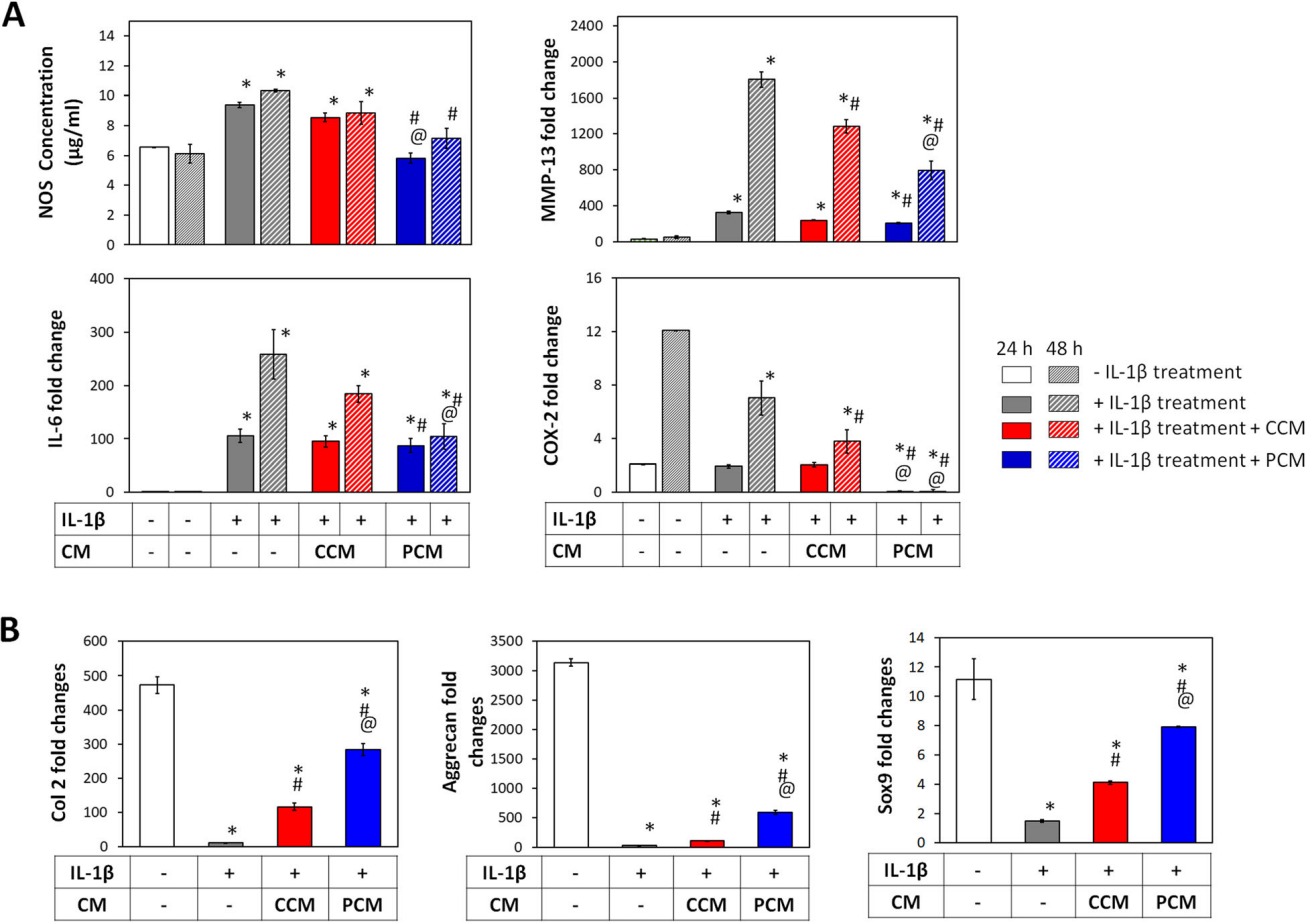
Effect of PEMF-induced conditioned medium (CM) on inflamed MSCs pre- (**a**) and post- (**b**) chondrogenic induction. **a** Inflammation was induced in MSCs with 5 ng/ml IL-1β for 24 h before administration of CCM or PCM generated from 2D MSC cultures. Expression of inflammatory genes and NOS activity was assayed at 24 (plain bars) and 48 h (hatched bars) after CM treatment. **b** MSCs undergoing chondrogenesis in 3D pellet cultures were treated with 5 ng/ml IL-1β 24 h prior to the administration of CCM or PCM. Expression cartilaginous markers was analyzed after 7 days of differentiation, normalized to GAPDH and presented as fold-changes relative to levels in undifferentiated MSCs. Data shown represent means ± SD, *n* = 6 from 2 independent experiments. * denotes significant differences compared to the non-inflamed controls (no IL-1β treatment), # denotes significant differences compared to the inflammation controls (IL-1β alone treatment), and @ denotes significant differences compared to respective CCM. Col 2 = type II collagen; COX-2 = cycloxigenase-2; IL-1β = interleukin-1β; IL-6 = interleukin-6; MMP-13 = metalloproteinase 13; NOS = *nitric oxide synthase*; Sox9 = SRY-Box 9

Similar to chondrocytes, IL-1β treatment elicited a state of inflammation in MSCs that was evidenced by upregulations in IL-6, MMP-13, COX-2, and nitric oxide synthase (NOS) (Fig. 8a). By contrast, MSCs were overall more resistant to developing IL-1β-induced inflammation as appreciable levels of inflammatory markers were generally only achieved after 48 h. Reductions in MMP-13 and COX-2 were observed with both CCM and PCM administration at 24 and 48 h post-administration with overall greater suppression observed with PCM. The levels of COX-2 showed significant reductions at both 24 and 48 h upon administration of PCM, particularly with reference to non-inflammatory controls (no IL-1β treatment). IL-6 levels were suppressed most strongly 48 h after administration of PCM relative to the inflammatory control (IL-1β treatment). PCM administration also produced significant suppressions of IL-1β-induced NOS activity at both 24 and 48 h, whereas CCM showed no effect at either time point. Notably, the level of NOS activity observed in response to PCM administration at 48 h was identical to that in the non-inflammatory control, indicating complete normalization of basal NOS activity.

The effects of PCM over MSC chondrogenesis were also examined under conditions of induced inflammation. IL-1β treatment suppressed the expression of the chondrogenic markers, Col 2, aggrecan, and Sox9 (Fig. 8b). Partial rescue of Col 2 (> 9-fold and 20-fold), aggrecan (> 3-fold and 15-fold) and Sox9 (> 2.5-fold and 5.5-fold) was achieved in samples supplemented with either CCM or PCM, respectively, compared to the inflammatory controls (IL-1β treatment). As noted, PCM consistently produced greater levels of protection than CCM. PEMF treatment hence was capable of enhancing MSC-derived paracrine factors capable of attenuating cellular inflammation, partially reinstating MSC chondrogenesis.

### Effect of PCM on chondrocyte and MSC apoptosis

**Fig. 9:**
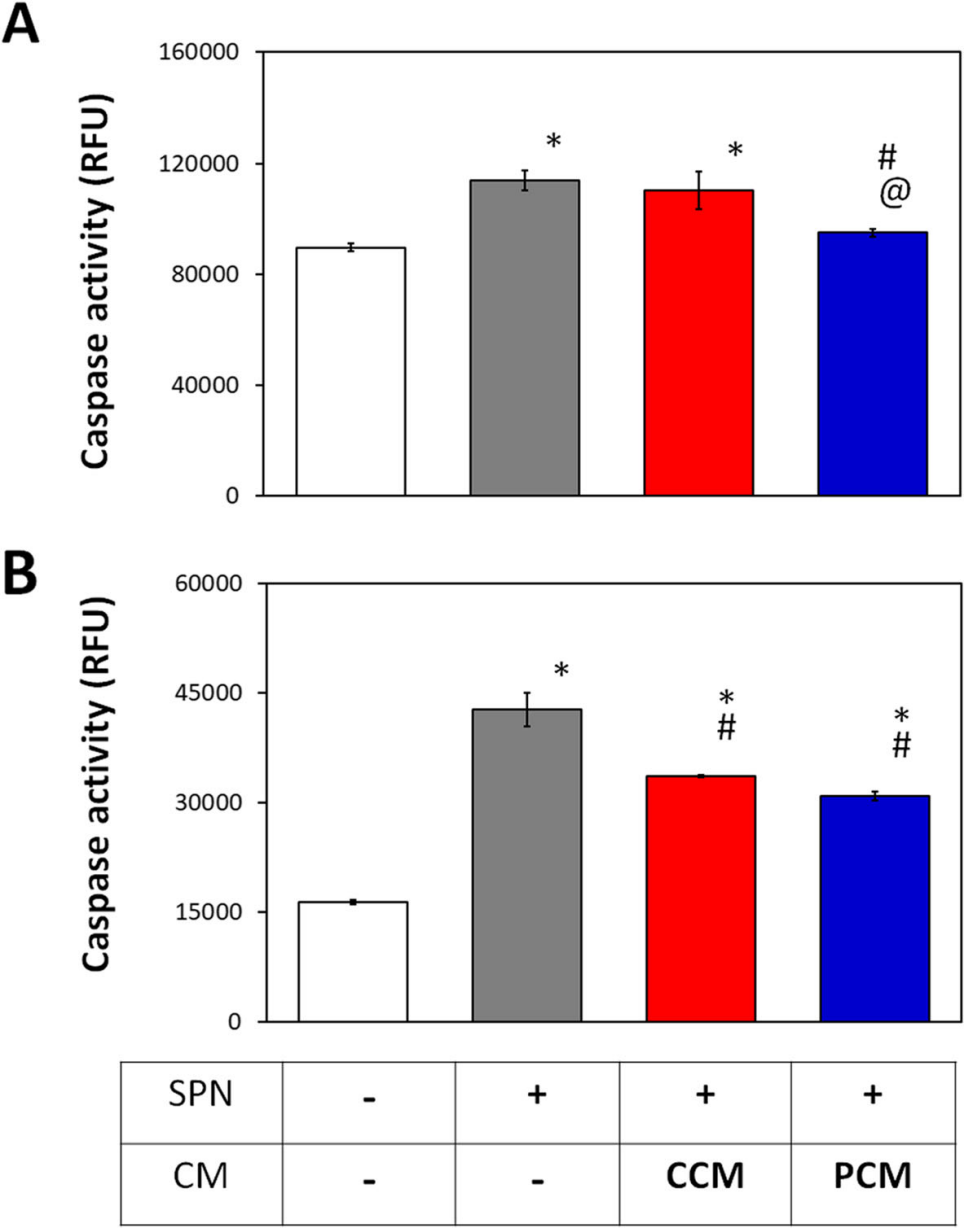
Effect of PEMF-induced conditioned medium (CM) on the apoptotic status of MSCs and chondrocytes. Chondrocytes (**a**) or MSCs (**b**) were treated with Staurosporin (SPN; 200 nM) in conjunction with supplementation with either CCM or PCM for 2 h. Apoptotic activity was determined by Caspase 3/7 activity and was presented as relative fluorescence units (RFU). Data shown represent means ± SD, *n* = 6 from 2 independent experiments. * denote significant differences compared to no Staurosporin treatment; # denotes significant differences compared to Staurosporin treatment alone and @ denotes significant differences relative to CCM

We have previously shown that brief (10 min) exposure to PEMFs was capable of attenuating basal apoptosis in myoblasts during early myogenesis [35]. We evaluated the potential of 2D MSC-generated CM to modulate apoptosis in chondrocytes and MSCs subjected to Staurosporin (200 nM). Basal caspase activity was elevated in control chondrocytes reflecting the elevated resting inflammation demonstrated previously (Fig. 7) and could be further augmented with exposure to Staurosporin. PCM significantly suppressed caspase activity in chondrocytes after Staurosporin treatment, effectively normalizing basal apoptosis levels (Fig. 9a). By contrast, no antiapoptotic effect was observed in chondrocytes administered CCM relative to the Staurosporin-treated control. MSCs in which apoptosis was induced with Staurosporin and supplemented with either CCM or PCM exhibited a comparable and significant decrease in caspase activity relative to the Staurosporin-treated control (Fig. 9b). PEMF treatment hence potentiates the paracrine-mediated attenuation of apoptosis of MSC CM in chondrocytes that could ultimately translate to improved regenerative responses.

### Expression and secretion of paracrine factors modulating differentiation, proliferation, migration, and inflammation from PEMF-treated MSCs

**Fig. 10:**
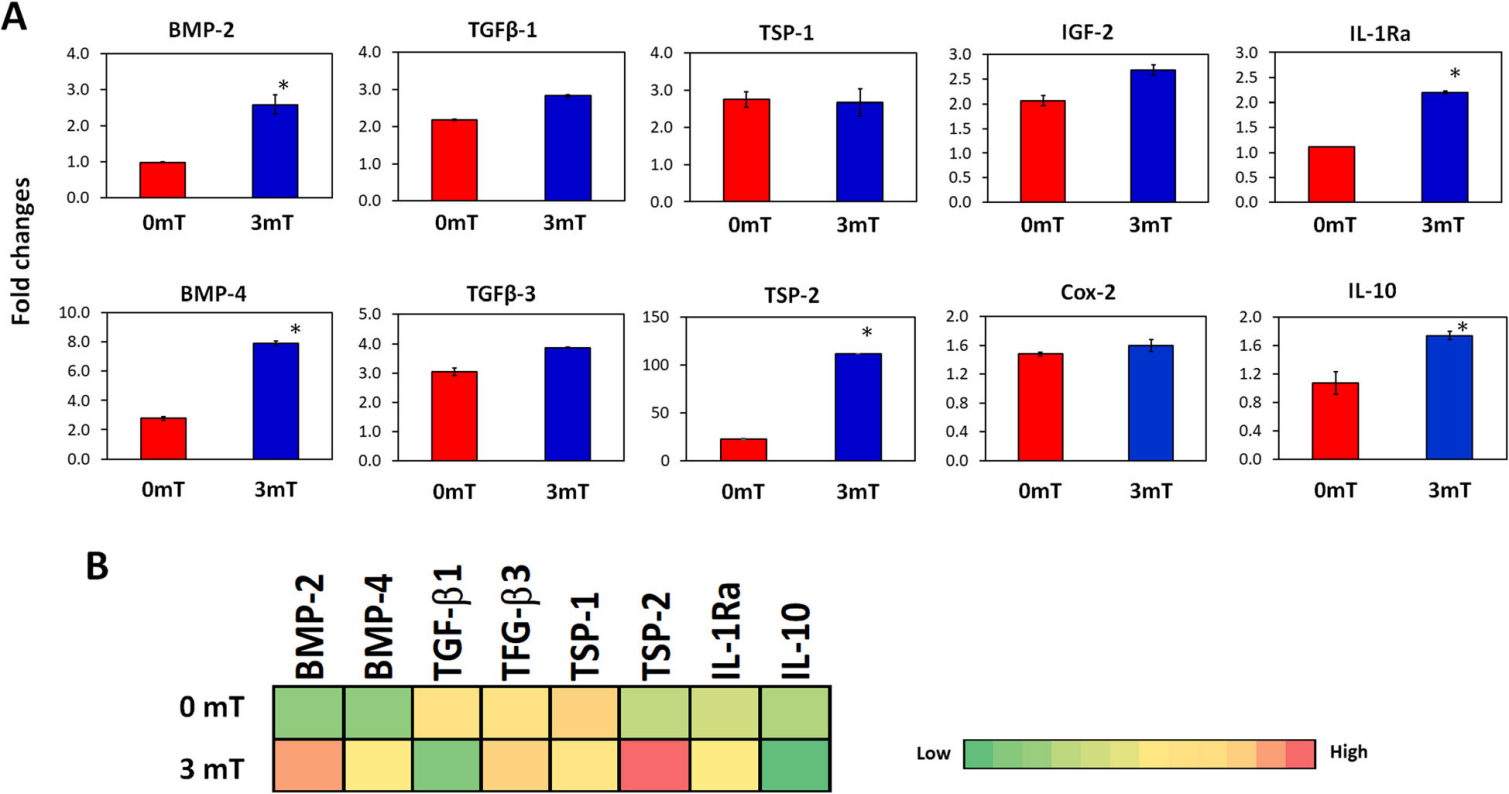
PEMF exposure modulates the secretion of MSC-derived paracrine factors. **a** 2D cultures of MSCs without (0 mT) and with PEMF exposure at 3 mT for 10 min were subjected to real-time PCR analysis after 24 h. Data shown represents means ± SD, *n* = 6 from 3 independent experiments. **b** Heatmap generated from an antibody microarray performed on the CM showing differences in the secretion profile of paracrine factors from MSCs. BMP = bone morphogenetic protein; COX-2 = cycloxigenase-2; IGF = insulin-like growth factor; IL-1ra: interleukin 1 receptor antagonist; TGF = transforming growth factor; TSP = thrombospondin

The ability of PEMFs to influence the production and release of factors associated to chondrogenic differentiation, proliferation, migration, and inflammatory modulation was examined from 2D cultures of MSCs. Factors examined included bone morphogenic protein (BMP), transforming growth factor (TGF), thrombospondin (TSP), insulin-like growth factor (IGF), COX-2, IL-10, and interleukin 1 receptor antagonist (IL-1ra). The expression of BMP2, BMP4, TSP-2, IL-1ra, and IL-10 were upregulated by ~ 2-fold, or greater, 24 h after PEMF treatment, relative to non-exposed MSCs. TGFβ1, TGFβ3, IGF-2, and COX-2 exhibited less significant increases, whereas TSP-1 showed no change in expression (Fig. 10a).

Antibody arrays were used to characterize the secretion profile of the MSC CM. PEMF exposure at 3 mT promoted the secretion of BMP-2, BMP-4, TSP-2, and IL-1ra (Fig. 10b), consistent with observed increases in gene expression (Fig. 10a). By contrast, the protein secretion of TGFβ1 and IL10 was dampened by PEMF treatment, while TGFβ3 and TSP-1 secretion was not affected, reflecting transcriptional to translation temporal disparity.

## Discussion

MSCs secrete a plethora of bioactive factors, which through paracrine means, synchronize the regenerative responses of neighboring cellular communities. For clinical exploitation, strategies to selectively modulate the secretory function of MSCs have been advanced and include subjecting MSCs to a variety of micro-environmental biochemical and mechanical cues, as well as biophysical perturbations [24, 25, 50]. We previously showed that MSC chondrogenic differentiation in 3D pellet cultures could be enhanced by brief exposure to low amplitude and frequency PEMFs administered for 10 min at an optimal amplitude of ~ 2 mT [34]. The involvement of transient receptor potential cation (TRP) channels and downstream Ca^2+^ signaling were implicated in mediating the effects of PEMF exposure. PEMFs were subsequently shown to preferentially activate a TRPC1-mitochondrial axis important for in vitro myogenesis and underlying mitochondrial adaptation to oxidative stress via a process of magnetic mitohormesis [35]. Accordingly, activation of mitochondrial respiration is known to activate the muscular secretome [33].

In this study, we examined the effects of brief PEMF exposure on the MSC secretome and its ability to promote MSC-induced chondrogenesis and chondrocyte cartilage tissue formation. The results demonstrated that the capacity of PEMFs to induce MSC chondrogenesis is contributed to by PEMF modulation of MSC secretome. As such, exchange of the bathing medium 24 h post-PEMF exposure compromised chondrogenesis, clearly indicating the necessity of released paracrine factors (Fig. 2). Harvested CM from PEMF-treated MSCs (PCM) was moreover capable of conferring chondrogenic enhancement onto naïve MSCs and, furthermore, demonstrated synergy when combined with direct PEMF exposure of MSCs. Notably, the PEMF parameters best suited for the production of chondrogenic MSC-PCM from 3D pellets was 2 mT for 10 min (Fig. 3), identical to the exposure parameters previously determined most efficacious at promoting 3D MSC chondrogenesis in response to direct exposure [34]. PCM generated under this exposure paradigm rendered significant chondrogenic enhancement compared to CCM obtained from non-exposed MSCs. Furthermore, the observed PCM-mediated increase in the expression of cartilaginous matrix was accompanied by a downregulation of hypertrophic markers, Col 10, ALP, and MMP13. The pro-chondrogenic effect of PCM was also evident in the context of chondrocyte redifferentiation, promoting cartilage formation with superior hyaline phenotype (Fig. 5). Taken together, our data demonstrates that MSCs subjected to chondrogenic induction under conditions of 3D culture respond to PEMF stimulation with secretome modulation that could provide both autocrine and paracrine anabolic effects with ultimate relevance to the in vivo articular scenario.

Components of the MSC secretome, in the form of CM or as isolated extracellular vesicles (EVs), have demonstrated therapeutic potential in cases of osteochondral lesions [13, 14] or in osteoarthritis animal models [15, 16]. The therapeutic effect of the MSC secretome has been largely attributed to the multifaceted stimulation of chondrocyte proliferation, migration, cartilaginous ECM generation, and the attenuation of the inflammatory and apoptotic microenvironment associated with injury or joint degeneration [14–16, 51, 52]. The demonstration that the delivery of MSC secretory products was efficacious in cartilage regeneration revealed the possibility of using MSC-derived secretory products as a cell-free therapeutic for joint injury and osteoarthritis. Notably, CM harvested from MSCs in conventional 2D tissue culture had similar chondrogenic potency as 3D PCM in terms of cartilage ECM formation and the capacity to reduce hypertrophic and fibrocartilage development for both MSCs and chondrocytes, although responsive to a distinct, yet higher (3 mT for 10 min), electromagnetic efficacy window (Figs. 4 and 5). Chondrocyte migration had been previously reported to be stimulated by MSC-derived EVs [14, 51]. Here we show that migration was also enhanced for both chondrocytes and MSCs by PCM (Fig. 6), suggesting the potential for PCM to chemotactically attract chondrocytes or MSCs to the vicinity of an articular injury to promote regeneration.

Following cartilage injury or during osteoarthritis, the expression of inflammatory cytokines and catabolic factors (e.g., matrix metalloproteinases) are upregulated, perpetuating inflammation, cartilage matrix degradation, and chondrocyte apoptosis [53]. MSC-derived EVs have been previously demonstrated to inhibit the adverse effects of inflammatory cytokines on cartilage homeostasis [14–16, 51, 52]. Here we show that the MSC-derived CMs are generally protective against cellular inflammation reflected by suppressions of chondrocyte NOS activity, inflammatory markers (IL-6 and COX-2), and catabolic proteinases (MMP13) (Fig. 7). Significantly, the protective impact of PCM generated from PEMF-exposed MSCs was even greater as revealed by stronger suppressions of NOS activity, preservation of chondrocyte anabolic activities, indicated by upregulating severely suppressed cartilage ECM genes expression (Fig. 7), and rescuing the Staurosporin-induced chondrocytes apoptosis (Fig. 9), an effect not observed with non-PEMFed control CM (CCM).

The protection conferred by PCM may hence potentially be extended to an inflamed scenario within the articular environment. Resident MSCs in the joint cavity, arising from either the synovium or bone marrow, in the case of microdrilling/microfracture, have been shown to participate in cartilage regeneration, either by engraftment or as endogenous providers of secreted trophic factors [54, 55]. The regenerative efficacy of resident MSCs, however, would be influenced by the inflammatory environment within the joint. Under conditions of induced inflammation, MSCs experienced increased NOS activity, elevated expressions of IL-6, Cox-2, and MMP13, and attenuated chondrogenic differentiation, degenerative conditions that were effectively suppressed with PCM (Fig. 8) and could potentially be translated to the in vivo scenario. Our results indicate that the brief exposure of MSCs to low-amplitude PEMFs heightens the anti-inflammatory potential of their secretome, alluding to an enhanced therapeutic application to attenuate cartilage damage and restore MSC regenerative capacity in an inflamed articular environment.

MSCs secrete trophic factors including IGF-1, PDGF, FGF, VEGF, and members of the TGFβ superfamily in response to environmental cues or stimuli [18, 19]. Accordingly, PEMF-based enhancement of MSC differentiation has been previously linked with the expression and secretion of the TGF and BMP families [36–38]. Moreover, the capacity of PCM to significantly augment cartilaginous ECM production, despite the conventional presence of a relatively high dose of exogenous TGFβ3 (10 ng/ml), alludes to the presence of additional factor(s) necessary for chondrogenic induction. Accordingly, we did not detect significant differences in the expression of TGFβ between PEMFed and non-PEMFed MSCs (Fig. 10) and moreover, TGFβ1 secretion was instead reduced in the PCM. By contrast, PEMF exposure of MSCs produced ≥ 2-fold increases in the expressions of BMP2, BMP4, TSP2, and IL1ra that were further corroborated at the protein level using antibody arrays. BMP2 [56], BMP4 [57], and TSP2 [58–60] have been previously implicated in chondrogenic induction. Indeed, autocrine action of TSP-2 from umbilical cord MSCs has been shown to suppress hypertrophic phenotype development [60] and could possibly account for our observed suppression of MSC and chondrocyte hypertrophy in response to administration of PCM. BMP2 and BMP4, on the other hand, are implicated as chemotactic agents for MSCs [61] and could be responsible for the enhanced migratory effect we observed exerted by PCM on chondrocytes and MSCs. Finally, IL-1ra is the biological inhibitor of IL-1β, associated with the severity of diseased cartilage degeneration [62] and has been demonstrated to reduce cartilage catabolism [63]. Nonetheless, the screening and confirmation of candidate factors participating in the chondrogenic enhancement observed with PCM is far from complete. In addition, the possible role of exosomal CD73-mediated adenosine activation of AKT, ERK, and AMPK signaling [14, 64] and the participation of exosomal microRNAs [65, 66] that have been previously implicated in regulating chondrocyte anabolic activity and cartilage degradation have yet to be examined. Further work will be required to fully decipher the paracrine mechanisms responsible for the enhanced anabolic and protective effect observed here in response to PCM administration, including the examination of the contribution of EVs to our reported PEMF-mediated secretome responses.

PEMF stimulation has been previously shown by others to exert anti-inflammatory effects by activating adenosine receptors [41, 44], while modulating intracellular calcium and activating the mechanotransduction FAK/Rho GTPases signaling pathways to induce MSC migration [67]. PEMF exposure was also shown to activate a calcium-mitochondrial axis stimulating mitochondrial respiration and promoting both mitochondriogenesis and myogenesis as well as reducing basal apoptosis and increasing telomere length via a process of adaptive magnetic mitohormesis [35]. Uniting these seemingly disparate responses is the finding that mechanical stimulation enhances mitochondrial ROS formation [30]. Accordingly, the generation of mitochondrial ROS has been shown to stimulate the secretome activity and is required for the development of the cellular and organismal adaptations against cellular inflammation [33]. It thus appears that mechanotransduction and mitochondrial respiration act upstream of secretome-mediated anti-inflammatory responses and appear to be modulated by magnetic fields as reported here. Subsequent studies will examine the mitochondrial contributions to the MSC secretome modulation induced by PEMF exposure.

We have previously demonstrated cell type-specific electromagnetic efficacy windows for PEMF-induced cytotoxicity of breast cancer cells [68], myogenic induction [35], or MSC-based chondrogenic induction [34]. Here, we extend the biophysical criteria defining the electromagnetic efficacy window by showing that the culturing microenvironment could modulate magnetic sensitivity of MSCs and downstream secretome activity. 2D and 3D MSC culture platforms exhibited distinct sensitivities to PEMF stimulation; a predominance of cell-cell interactions in the 3D configuration gave rise to higher magnetic sensitivity with lower activation window. The optimum PEMF amplitude to produce PCM with enhanced biological efficacy from 2D and 3D MSC cultures was 3 mT and 2 mT, respectively. MSCs grown on 2D TCP adopted fibroblastic morphologies and exhibited a predominance of cell-substrate interactions, whereas MSCs in 3D exhibited rounded morphologies and were largely dominated by cell-cell contacts. These distinct mechano-environmental scenarios likely elicited different mechanotransduction responses, which in turn converged with, or differently conditioned, the cellular response to magnetic field stimulation. For instance, cellular aggregation has been shown to trigger cadherin-related cell-cell interactions, which in itself augments MSC-dependent wound healing, myogenic, anti-inflammatory, and angiogenic responses [27–29, 48]. It is thus possible that altering the cellular mechanical environment would influence MSC secretome composition and function as well as response and sensitivity to magnetic field exposure. Despite differences in PEMF sensitivities, similar levels of chondrogenic outcome and cartilage formation resulted from either 2D or 3D PCM administration (Figs. 3, 4, and 5). It remains to be resolved, however, whether any differences exist in the exact secretome profile of the PCM (or CCM) harvested from either platform.

## Conclusions

We provide evidence that brief exposure to low amplitude PEMFs enhanced the ability of MSCs to produce and secrete paracrine factors capable of promoting cartilage regeneration as well as protecting against adverse inflammatory conditions. Furthermore, this report highlights the importance of optimizing PEMF exposure parameters for MSCs subjected to different culturing conditions. Collectively, our results indicate that PEMF stimulation could augment the production and release of the MSC paracrine repertoire for the ultimate enhancement of cartilage regeneration.

## Additional file

Additional file 1: **Table S1.** A) Human PCR primer sequences. B) Porcine primer sequence (DOCX 13 kb)

## Abbreviations

ALP: Alkaline phosphatase
BMP2: Bone morphogenetic protein 2
BMP4: Bone morphogenetic protein 4
cAMP: Cyclic adenosine monophosphate
CM: Conditioned medium
CCM: Non-PEMFed CM
Col 2: Type II Collagen
Col 1: Type I Collagen
Col 10: Type X Collagen
COX-2: Cycloxigenase-2
ECM: Extracellular matrix
EVs: Extracellular vesicles
IGF: Insulin-like growth factor
IL-1β: Interleukin-1beta
IL-6: Interleukin 6
IL-10: Interleukin 10
IL-1ra: Interleukin 1 receptor antagonist
MSC: Mesenchymal stem cell
MMP13: Metalloproteinase 13
NOS: *Nitric oxide synthase*
PCM: PEMFed generated CM
PEMF: Pulsed electromagnetic field
PGE2: Prostaglandin E2
PCR: Polymerase chain reaction
sGAG: Sulfated glycosaminoglycan
Sox9: SRY-Box 9
TGF: Transforming growth factor
TRP: Transient receptor potential
TSP: Thrombospondin
