# Supplementary material for: Pulsed electromagnetic fields potentiate the paracrine function of mesenchymal stem cells for cartilage regeneration
Source: Stem Cell Res Ther. 2020 Feb 3;11:46. doi: 10.1186/s13287-020-1566-5 (PMC6998094; doi:10.1186/s13287-020-1566-5)
Supplement: Supplementary file 1 — Table S1. A) Human PCR primer sequences. B) Porcine primer sequence (DOCX 13 kb) [file 13287_2020_1566_MOESM1_ESM.docx]

**Table S1**.

A) Human PCR primer sequences.

| **Gene** | **Forward Primer Sequence** | **Reverse Primer Sequence** |
| --- | --- | --- |
| GAPDH | ATGGGGAAGGTGAAGGTCG | TAAAAGCAG CCCTGGTGACC |
| Aggrecan | ACTTCCGCTGGTCAGATGGA | TCTCGTGCCAGATCATCACC |
| Sox9 | AGTACCCGCACTTGCACAA | CTCGTTCAGAAGTCTCCAGAGCTT |
| Col 1 | CAGCCGCTTCACCTACAGC | TTTTGTATTCAATCACTGTCTTGCC |
| Col 2 | GGCAATAGCAGGTTCACGTACA | CGATAACAGTCTTGCCCCACTT |
| Col 10 | CAAGGCACCATCTCCAGGAA | AAAGGGTATTTGTGGCAGCATATT |
| ALP | GGGGGTGGCCGGAAATACAT | GGGGGCCAGACCAAAGATAG |
| MMP13 | TCCTCTTCTTGAGCTGGACTCATT | CGCTCTGCAAACTGGAGGTC |
| COX-2 | CAGCACTTCACGCATCAGTTT | GCGCAGTTTACGCTGTCTA |
| IL-6 | GATGGCTGAAAAAGATGGATGC | TGGTTGGGTCAGGGGTGGTT |
| IL-10 | GGTTGCCAAGCCTTGTCTGA | AGGGAGTTCACATGCGCCT |
| IL-1ra | AAGATGTGCCTGTCCTGTGTCAA | GTTCTCGCTCAGGTCAGTGATGTTA |
| BMP-2 | ATGGATTCGTGGTGGAAGTG | GTGGAGTTCAGATGATCAGC |
| BMP-4 | ACCACGAAGAACATCTGGAGAA | TGCTTTTCGTTTCCTCTTTAACCT |
| TGFβ-1 | CGCATCCTAGACCCTTTCTCCTC | GGTGTCTCAGTATCCCACGGAAAT |
| TGFβ-3 | GGTTTTCCGCTTCAATGTGT | TATAGCGCTGTTTGGCAATG |
| IGF-2 | GTGGCATCGTTGAGGAGTG | CACGTCCCTCTCGGACTTG |

TSP-1 and TSP-2 primers were obtained from Bio-Rad, Unique Assay ID: dHsaEG5009404 and qHsaCID0011987, respectively.

B) Porcine primer sequence

| **Gene** | **Forward Primer Sequence** | **Reverse Primer Sequence** |
| --- | --- | --- |
| GAPDH | ATGGTGAAGGTCGGAGTGAA | AATGAAGGGGTCATTGATGG |
| Aggrecan | CCAGGGGCAAATGTAAAGG | CATCACCGAGGGTGAAGC |
| Col I | CCAGTCACCTGCGTACAGAA | ACGTCATCGCACAACACATT |
| Col II | TGAGAGGTCTTCCTGGCAAA | GAAGTCCCTGGAAGCCAGAT |
| Col10 | TGCTGCTGCTATTGTCCTTG | TGAAGAACTGTGCCTTGGTG |
| ALP | GGAATTCCCAGACAGCACAAA | AAGAGCATGTGCGGGAAAGT |
| MMP13 | CTTGTTTCTTGTTGCTGCCC | GTGGGTCTTCATCTCCTG |
| IL-6 | GACAAAGCCACCACCCCTAA | CTCGTTCTGTGACTGCAGCTTATC |
| COX-2 | TTCAACCAGCAATTCCAATACCA | GAAGGCGTCAGGCAGAAG |
